# “Tell me what is ‘better’!” How medical students experience feedback, through the lens of self-regulatory learning

**DOI:** 10.1186/s12909-023-04842-9

**Published:** 2023-11-22

**Authors:** Muirne Spooner, James Larkin, Siaw Cheok Liew, Mohamed Hasif Jaafar, Samuel McConkey, Teresa Pawlikowska

**Affiliations:** 1 Health Professions Education Centre, Royal College of Surgeons in Ireland University of Medicine and Health Sciences, Dublin, Ireland; 2 Department of General Practice, Royal College of Surgeons in Ireland University of Medicine and Health Sciences, Dublin, Ireland; 3 Department of General Practice, Perdana University Serdang, Selangor, Malaysia; 4 Department of International Health & Tropical Medicine, Royal College of Surgeons in Ireland University of Medicine and Health Sciences, Dublin, Ireland

**Keywords:** Medical students, Feedback response, Feedback use, Emotions, Negative feedback, Self-regulatory learning, Feedback engagement

## Abstract

**Introduction:**

While feedback aims to support learning, students frequently struggle to use it. In studying feedback responses there is a gap in explaining them in relation to learning theory. This study explores how feedback experiences influence medical students’ self-regulation of learning.

**Methods:**

Final-year medical students across three campuses (Ireland, Bahrain and Malaysia) were invited to share experiences of feedback in individual semi-structured interviews. The data were thematically analysed and explored through the lens of self-regulatory learning theory (SRL).

**Results:**

Feedback interacts with learners’ knowledge and beliefs about themselves and about learning. They use feedback to change both their cognitive and behavioural learning strategies, but how they choose which feedback to implement is complex. They struggle to generate learning strategies and expect teachers to make sense of the “how” in addition to the “what”” in planning future learning. Even when not actioned, learners spend time with feedback and it influences future learning.

**Conclusion:**

By exploring our findings through the lens of self-regulation learning, we advance conceptual understanding of feedback responses. Learners’ ability to generate “next steps” may be overestimated. When feedback causes negative emotions, energy is diverted from learning to processing distress. Perceived non-implementation of feedback should not be confused with ignoring it; feedback that is not actioned often impacts learning.

**Supplementary Information:**

The online version contains supplementary material available at 10.1186/s12909-023-04842-9.

## Background

Feedback has potential to support learners through transformational change [1, 2]. When effective, it is one of the most influential factors in improving academic achievement and promoting learning [3]. However, feedback and feedback processes are variably interpreted [4] and poorly executed, i.e. explicit messaging with developmental planning does not occur [5]. While learners in higher education value feedback, they also consistently communicate dissatisfaction with feedback [6], and frequently do not use it [7–10].

Literature has traditionally reported teacher-centred unidirectional feedback, which has been reconceptualised in health professional education [11, 12]. Learners are now considered active participants who collaborate in their developmental planning. This re-focus on feedback uptake has identified attributes of learner proactive recipience as fundamental to its success: SAGE (self-assessment, assessment literacy, goal-setting and self-regulation, engagement and motivation). Feedback literacy, defined as “the understandings, capacities and dispositions needed to make sense of information and use it to enhance work or learning strategies”, positions the learner as engaged and competent in making sense of, and implementing feedback [13].

Self-regulatory learning (SRL) theory is a well-established theory with origins in psychology, education and sociology. It is a style of engaging with tasks which is characterised by setting goals, selecting strategies, balancing cost and effect, monitoring, adapting and amending behaviours. It proposes a holistic understanding of learning, with cognitive, metacognitive, behavioural, motivational and emotional influences Key aspects of SRL include active learners who draw on existing knowledge and beliefs and monitor outcomes to recalibrate accordingly [14]. It is therefore a helpful model to understand how learners take up learning information such as feedback.

A number of SRL models exist; this study will employ the shared concepts emphasised across three seminal models [15–17]. Firstly, SRL takes a situated learning approach with an emphasis on context. This aligns with established findings that feedback responses cannot be separated from the context in which they occur [18]. Secondly, self-regulation is characterised by goal-directedness, with contemporary teaching focussing on feedback that effects change for learning gain. “Closing the loop” describes moving from the learner’s current standard to a higher level via actively implementing feedback [19]. “Coaching for change” relates to the R2C2 model of feedback conversations, whereby coaching is part of a collaborative bi-directional feedback interaction resulting in an agreed developmental plan [20]. SRL emphasises that learner’s behavioural changes in response to learning information relate to their motivations and cognition. Feedback responses are often linked to motivation with a growth mind-set and applying cognitive ability to implement it [21]. Another key feature is its recursive nature: the self-regulated learner constantly checks, re-calibrates and tweaks learning strategies. Finally, it is a dynamic process which alters with each iteration and with differing contexts.

Proactive recipience has developed understanding of the factors required for learner engagement and uptake of feedback. Feedback literacy builds on this in centring the learner in the act of using feedback. Self-regulated learning (SRL) theory offers another lens to delve in to the dynamic strategies that play out in the learner in any given feedback interaction. It brings together aspects of these concepts under one framework. The SAGE taxonomy (self-assessment, assessment literacy, self-regulation, engagement and motivation) indicates self-efficacy and enthusiasm are needed to for proactive feedback recipience, which parallels SRL’s assertion that self-belief and motivation influence how learning information (such as feedback) is implemented in future learning. The concepts of “making judgments” and “managing emotions” in feedback literacy overlap with SRL aspects of metacognitive awareness and affective regulation, respectively. SRL may be useful therefore as an overarching framework to consider how all these factors interact with a holistic approach. By applying the lens of SRL, we aim to explore how self-regulatory activity interacts with incorporating feedback for improved learning. We aim to examine sub-processes that interact between affective, cognitive, behavioural and contextual factors. It allows us to consider the feedback interaction in terms of if and how it activates SRL, and at which sub-areas. Hence our research question:

In what ways do learners’ experiences of feedback relate to self-regulation of learning?

## Methods

### Study design and methodological orientation

This study was approached from a relativist ontology [22], where feedback is conceptualised as a social act, constructed by participants, circumstance and environmental factors. We consider feedback a dynamic concept which changes meaning depending on the individual and their particular context, e.g. grades, face-to-face appraisals, written reports. The researcher epistemology regarding feedback is socio-constructivist; what feedback means is an expression of the values and beliefs of the participants in the feedback activity and are embedded in the specific cultural and social context. Our methodological approach is exploratory using qualitative methods in interpreting the lived experience of feedback for medical students. This study is reported in accordance with the consolidated criteria for reporting qualitative research (COREQ) (Appendix B) [23].

### Participants

This study was undertaken with final-year medical students in the RCSI (Royal College of Surgeons in Ireland) University of Medicine and Health Sciences. Student participants from the three campuses in Ireland, Bahrain and Malaysia were invited to participate. All students were introduced to the study with a presentation explaining background to the study and research questions. They are provided with assessment and feedback information for each year of study, but not provided with directed training on how to use feedback.

### Researchers

MS is a senior lecturer who regularly engages in feedback with the final-year medical students. TP is professor emerita of the Health Professions Education Centre. MS and TP are involved in feedback research and have published a systematic review on feedback responses. JL and MHJ are researchers at the institution who do not have a role in undergraduate education. Although some authors (SCL, SM, TP, MS, TP) are involved in education, the interviewer at each site (JL-Ireland, MS- Bahrain, MHJ- Malaysia) was not involved in student assessment and learning, in recognition of principles of interpersonal reflexivity and acknowledging the power dynamics that could influence participants [24]. Team discussions facilitated diverse narratives being captured in our interpretation [25, 26].

### Recruitment and data collection

Our institution is a transnational, multi-campus medical school and our student population is diverse, with students from 101 nationalities [27]. Ethics approval was sought and received at all sites. We conducted individual semi-structured interviews to explore students’ feedback experiences. We used the theoretical framework of SRL to design the interview guide (Appendix A). We conducted pilot interviews at each site, which were recorded. MS listened to these and reviewed the transcripts, then refined the interview protocol with TP and JL. On commencing each interview, participants were provided with examples of feedback formats and a broad definition. Participants were encouraged to describe their experiences, including examples of why and how they use feedback. We did not specify particular feedback events, so participants were free to discuss any experiences. We sampled for maximal diversity for nationality, gender and site of study. Data analysis commenced after five interviews and continued iteratively until it was considered that information power was sufficient [28].

### Data processing

The interviews in Ireland were done in person, those in Bahrain and Perdana were conducted online. All interviews were recorded and transcribed initially using transcription software (Otter.ai). Transcriptions were edited for accuracy, by comparing with audio recordings. All identifiable information was anonymised prior to any coding. Original recordings were subsequently destroyed.

### Data analysis

We chose template analysis as a systematic approach to thematic analysis. It is useful when managing large data sets such as ours. It emphasises use of hierarchical coding with high level of structure. It also allows for a priori codes, codes developed before examining the current data [29]. This facilitated informing this analysis with findings from a previous systematic review of feedback responses [4]. We followed the six-stage process [30]:

1. Familiarisation with data

MS and TP read and re- read the transcripts.

2. Preliminary coding

A priori themes were suggested by MS based on feedback responses aligned to concepts of self-regulatory learning theory. MS then inductively screened for new codes initially on a selection of interviews representing each campus site, students of varying nationality and gender. Rather than coding line-by-line, each transcript was read and re-read to identify high-level themes and sub-themes relevant to the research questions.

3. Clustering

Newly identified themes were clustered with existing ones via visual mapping. A priori codes which were agreed to be unhelpful in the analysis were removed.

4. Producing an initial template

A draft template was developed from the clustering of high-level themes (MS).

5. Applying and Developing the template

As further iterative analysis and discussion ensued (MS, TP), the template was revised to a final agreed hierarchy of major themes and lower-level codes (MS).

6. Final Interpretation

Major themes were agreed by consensus (MS, TP) and the final template consisted of three overarching domains. We fed back our analysis to students for respondent validation, and no significant changes were required (MS) [31].

### Template evolution

Table 1 includes more information on evolution of the template. Initial a priori codes were derived from a previous systematic review on feedback responses. The second iteration of the template consisted of 3 overarching domains: the SRL processes activated by feedback specific to the Pintrich model of SRL [15]. MS and TP used the template to code transcripts to evaluate for themes, add new themes, delete or modify existing themes and re-order the hierarchy of themes and sub-themes.. This led to the development of the third template iteration, with a clarified focus on supportive and unsupportive feedback experiences as classifications under which sub-themes of the specifically identified SRL responses occurred. The final iteration applies three overarching domains of modulation of learning from integrating the experience type (supportive and unsupportive) and SRL responses as moderating factors of these modulations.

**Table 1:** Evolution of template

	First iteration	Second iteration	Third Iteration	Final Template
Actions Taken	Derived from previous review	Research group members coding of interviews	Multiple rounds of coding by MS/TP independently and then together	Application of la framework of learning modulation as a result of feedback
Description		Emphasis on identifying patterns of SRL in participants experiences	Separation based on leaner experience	Focus on three themes of learning modulation, with integration of other themes within these
Themes and Hierarchical Structure	**Responses to Feedback** Cognitive Behavioural Affective **Moderating factors** Supervisor characteristics Learner characteristics Message characteristics	C**ognitive** Locate progress Self-assess **Behavioural** Maintain/increase effort Change task techniques **Affective** Self-efficacy Confidence Task interest	**Negative experience** Emotions Motivation Self-efficacy Non-implementation of strategies Learning value decreased **Positive Experiences** Motivation Self-efficacy Adapt strategies	**Modulation of learning goals** 1. Motivation and effort investment 2. Reduction of negative emotions/stressors **Modulation of knowledge and beliefs** 1. Knowledge of self 2. Beliefs about learning/learning task **Modulation of Learning Strategies** 1. Strategy selection 2. Strategy adaptation *•* Behavioural *•* Cognitive *•* Disengagement

## Results

Fifty-seven student interviews were conducted (demographics in Table 2).

**Table 2:** Participant profile

Gender	Male	Female			
	26 (46%)	31(54%)			
No. of students in full class from each group	269 (52%)	245(48%)			
Medical Programme	Direct Entry	Graduate Entry			
	47 (82%)	10 (18%)			
No. of students in full class from each group	449(87%)	65 (13%)			
Average Age	25.1 (21–33)				
Place I Call Home^a^	Europe	Arab States	U.S. and Canada	South East Asia	Other
	8 (14.0%)	20 (35%)	14(25%)	9 (16%)	6 (11%)
No. of students in full class from each group	87(17%)	227 (44%)	100(20%)	69(13%)	31(6%)

^a^Students asked to name country/countries that they call “home” These were then grouped by geographical area

### Modulation of metacognitive knowledge and beliefs

SRL describes modulation of cognitive, affective and behavioural processes to achieve competence. Learners describe a number of ways in which feedback affects how they think about learning (cognitive modulation). They use feedback to self-evaluate and locate their ability against their peers. Occasional tension arose when self-evaluation did not align with feedback; however learners mostly indicate accepting well-intended critique after reflection:

“We knew that this teacher is a nice teacher who's trying to help us…So if they made a comment about how bad we were, we accept that because we know they're not trying to be harmful. They're just trying to help us. So we will accept that” (S10).

Feedback interactions negatively affected self-efficacy when derogatory language and attacks on their character were employed. They report feeling “humiliated” and “devastated” for long periods afterwards. They ruminate on this feedback and its implications of their ability.

Feedback modulates learners’ beliefs related to learning itself. Participants describe deeper engagement with a particular activity following a positive feedback encounter. They describe a renewed enthusiasm for learning in general and active feedback-seeking in future activities. Learners adjusted learning value downwards when feedback encounters were hostile. They describe feedback as “pointless” and talk about “walking away” from learning. Further examples are provided in Table 3.

**Table 3:** Modulation of knowledge and beliefs

Beliefs about self	Positive effect	It really gives it gives you an indication of where you are. (S1) Yeah, even if it wasn't sufficient, it validates the parts that were good (S50)
	Negative effect	You don't feel you're good enough, and you get anxious, and then after that, guilty (S18) You can be a little bit like, kicked in the face when you're like, “God, I'm really not doing that good” (S5)
Beliefs about Learning	Positive effect	I feel like more enthusiastic to attend tutorials, I feel more enthusiastic to do further reading on the topic. And then I probably because of doing further reading, like, have more of an interest in that topic (S36)
	Negative effect	It's kind of hard to show care about it, and that doesn't help you work when you know that it's gonna be like that *(vague feedback*) (S39)

### Modulation of learning goals

SRL theory acknowledges motivational and effective influences in learning. Our findings indicated feedback interacts with these factors in relation to learning goals. These came under two main themes: Motivation and effort investment and Protecting Emotions/Avoiding Stressors.

#### Motivation and effort investment

Supportive feedback experiences inspired enthusiasm in participants, particularly where the supervisor was.

“really engaged in wanting to see you improve and is passionate about, you know, helping you make those extra steps, it really motivates me to change” (S8).

Participants harnessed this by increasing time or effort on the learning activity. They re-calibrate targets, change study schedule, and add study activities. This happens even when their performance was sub-optimal: as long as the feedback experience was constructive, they invested more planning and studying. Participants commonly experience perfunctory feedback which negatively affects effort investment:

“(they are) just sort of providing critique, because it's expected, so I leave a feedback session being like, “why did I even bother showing up? Like, nothing useful happened there”…you know, feedback for the sake of giving feedback” (S8).

With hostile experiences, some students are demotivated, while others argue that “I'll feel ashamed and sad…But I will remember it…so better ashamed during the class, rather than you make mistakes in the exam” (S48).

#### Protecting emotions/avoiding stressors

Learners avoid feedback where they feel threatened. This stems from prior experiences they describe as “humiliating”, “embarrassing” and “aggressive”. They becomes fixated on escaping. This colours future learning where some go out of their way to avoid a repeat incident. One participant broke down in tears describing a feedback situation where they felt “he was picking on me a lot” and goes on to say they no longer actively seek feedback, “that’s why I'm scared to talk to the consultants because of that experience” (S11). This also manifested as non-attendance; avoiding specific supervisors or activities, e.g. presenting on wards. Further examples are provided in Table 4.

**Table 4:** Modulation of learning goals

Motivation/Effort Investment	Positive effect	It (feedback) makes us just concentrate, actually put more effort into improving ourselves (S40)
	Negative effect	It’s just very discouraging especially if the way it's phrased like puts you down because then it makes you feel like you're not working hard enough or doing your job or whatever,, and then instead of being motivated to work harder, you might be discouraged (S16)
Protecting emotions/Avoiding Stressors	You can, if it's upsetting, think about it later. Don't think about it now (S8) “I’m embarrassed, and I just want to leave” (S18)	

### Modulation of learning strategies

SRL requires the learner to monitor their progress within their context and adapt accordingly. In relation to feedback, our learners describe two key areas where how they design their learning was affected. These are broadly categorised under two themes: Selecting strategies and adapting strategies.

#### Selecting strategies

Learners expressed difficulty translating feedback in to learning strategies. They favoured feedback which gave clear “next steps”. They comment that:

“you'll know what you need to do but you don't know how” (S50).

“Good” feedback meant when supervisors suggested how to change rather than just what needed work. A learner explains that feedback:

“helped me to concentrate on important points…And if he didn't give me that advice, I wouldn't actually go to the wards. And so that was very positive because it really directed me towards exactly what I need to do to improve” (S4).

They find it challenging to select strategies to use feedback which identified developmental areas, without explicit direction in how to address these. Some of this came from vague comments:

“if you give me feedback, like "everything's Okay", what am I gonna do with it?” (S45).

They describe unsuccessful attempts to gain clarity, lead to abandoning this strategy:

“I just stopped asking questions …I would live with the teachers in secondary school…and ask everything until we both were on the same page… Here, you can ask a basic question and you don’t get a proper response… So I just stopped asking, which is awful. Because now I don't understand lots of things. I have no one to ask” S26.

### Adapting strategies

We have divided these in to three themes: behavioural strategies, cognitive strategies and disengagement to manage emotions.

### Behavioural strategies

Participants provided many examples of how they incorporated feedback in to task-specific actions, e.g. changing technique in physical examination, adding questions in history-taking, presenting cases with tweaks to structure and prioritisation.

### Cognitive strategies

Feedback had more variable influences on cognitive learning strategies. Participants readily implemented task or activity-specific tweaks, e.g. using a specific app for pharmacology knowledge, redirecting study from written to verbal practice of presentations.

When more elaborate overhaul of their study strategy was suggested, responses were mixed. Some were open to adapt; others were uncomfortable changing reliable strategies, saying;

“I've found my studying habits to be pretty set in stone at this point…., they've done me well, up until now, if somebody told me to completely revamp everything, I would thank them for their advice and not actually implement it” (S4).

Learners regularly pick and choose which elements of feedback to incorporate in subsequent learning tactics:

“it's a lot about filtering the stuff that comes, whether it's good, whether it's bad, you know, if there's something worth using, to go with it” (S1).

### Disengagement

Learners disengage when feedback is perceived as hostile. Such encounters are frequent and have several manifestations—derogatory language, negative non-verbal communication (eye-rolling, sighing, admonishing tone), interrupting and shouting, making comments about character or ability to be a doctor. While the feedback message may be potentially useful, some consciously walk away:

“Whether what they said is true or not, if they phrased it in a way that I felt uncomfortable with, and I didn't appreciate, I will just be like "do you know what? Nah, thanks". If you're not going to be professional then, “Bye””S30.

Others feel overwhelmed in the moment:

“I shut down. I feel like a small child, I hate it” (S2).

While they feel they will not or cannot use this feedback, many learners spend a lot of time with it:

“I'll be like pondering over it…I'll be thinking about it quite some time ….if it's very very harsh, then it will remain forever” (S49).

Further examples are provided in Table 5.

**Table 5:** Modulation of learning strategies

Selecting Strategies	Struggle to select strategies independently	Until somebody tells you, “you need to do this”, or “do this instead,” I don't see how I could improve (S15) There’s not much I can do with a grade or do with what I didn't do well, if I have no idea how to fix it (S53) Don't just throw a comment and walk away. Show me! Tell me what is “better!” Don't tell me you could have done better. (S10)	
	Value feedback with explicit strategies	If they're saying “oh I think it'd be great if you did X”, I don't know, "if you were to have a more organized system, like if you have like a checklist mentally. Maybe you can try it.” That's great. That's a better way for me to organize my thoughts (S6)	
Adapting Strategies			
Behavioural strategies	I would have never improved if I had never met a doctor, who said "the history was terrible. And this is how you're going to fix it. This is exactly how you should say it."(S26)		
Cognitive strategies	Feedback requiring significant shifts	Adapt	“I would try my best to take their comments on board… I don't think any of my practices and techniques are, are kind of finite, to the point where they're not changeable. And I'm open to taking on new perspectives and different ways of doing things” (S35)
		Discard	“Studying on my own … I have that down…So that I would probably keep doing the way I do it. But when it comes to like, practical skills, I'm willing to change” (S11)
	Curating feedback	Trying to take a couple of nuggets from each tutorial and be like, "Oh, yeah actually, I can use that” (S37)	
Disengagement and management of emotions	I reach a point … when people are that way, ….whatever they say, I just don't take it in… you’re causing more harm than benefits (S10) I've seen lots of my friends quit because of poor feedback, like really negative feedback, really, at least temporarily (S48) you feel like it's not constructive and that's when it that you'd like shut down (S11)		

## Discussion

This study sought to explore how medical students’ experiences of feedback relate to self-regulation of learning. This is an important addition to the literature as it considers feedback responses from the perspective of future learning, underpinned by an established theoretical framework. The trustworthiness of our findings are strengthened by having conducted this research across three international sites with diverse student populations.

Our findings indicate that medical students often perceive feedback as something that happens to them, rather than with them. Contrasting with contemporary feedback research, we show that they often expect rater-led instruction in order to embed it in future learning. When learners use feedback to self-regulate, they change both metacognitive and behavioural learning strategies, but this is contextualised particularly by how feedback affects their emotions.

Learners described good feedback as explicit direction. This was further emphasised by examples of enacted task-specific feedback- they readily changed when told “how” or what”. Recent feedback models focus on learner-led planning [20, 32], with a focus on co-construction [33]; this is at odds with how our participants conceptualise it. It may be that models originating from postgraduate research overestimate the self-regulatory abilities of medical students. It is established that junior learners need more support in navigating learning and certain learner “types” can exist such as “effortful”, who need to be told what to do [34]. Therefore, we encourage educators to consider that postgraduate models may require modification to provide this more explicit support and gradual sharing of responsibility in undergraduate contexts.

Neither the concept of learners’ limitations in decoding feedback, nor feedback literacy are new [13, 35]. This study extends what is known about how learners are challenged by feedback. Medical students position themselves as recipients of information rather than active participants in making sense of what feedback means and how to enact it. Much work supports that even learners with high levels of self-regulation struggle to develop their learning strategies independently [36, 37]. Learners identify a need to take control of learning as they progress [38] but guidance through this progression appears critical.

Even when learners value feedback, they may choose not to use it. Cognitive strategies such as study habits were ingrained: only a few participants would consider changing them, even while acknowledging this could be worthwhile. Students’ beliefs about learning are relatively stable and influence how they interact with feedback [16, 39]. Second- level experiences potentially foster more absolutist stances [40] with medical students importing practices from prior learning [41]. It is possible that their pre-existing beliefs out-weigh feedback in affecting their learning strategies.

Unsupportive experiences create a conflict for students. While the learner may judge cognitively that the feedback has potential use, they either choose to disengage because of the hostile delivery, or they are incapable of engaging, because the emotional overwhelm distracts them from learning. Sargeant also identified that negative emotions are a barrier to accepting feedback [42]. Our findings suggest that negative emotions outweigh other considerations in guiding students’ feedback response. Previous work reports learners [43] experience tension when observed ability conflicts with the learner’s self-concept [43, 44]. Our findings are distinct from these prior findings. Feedback that highlights personal weaknesses disappoints but feedback that is hostile is distinct from this: it emotionally overloads and derails learning. We do not suggest that negative emotions are “bad”; we acknowledge their potential role in extrinsic motivation of learning [45, 46]. We propose that the emotions are not the problem, the lack of psychological safety is [47]. Students’ ability to engage with SRL is influenced by positive supervisor relationships [48], and the “uncertain” learner depends on supervisor relationships and a safe environment to self-regulate efficiently [34]. Employing the discourse of emotion in feedback as reflective practice, whereby supervisors are allies and facilitate reflection can reframe towards acceptance and constructive use [20, 49].

Efficient self-regulators make more use of feedback [2, 50]. Our findings suggest that “use” may not reflect all SRL responses. Use is often an externally observable outcome, whereas SRL reflects both the learner’s internal regulatory processes and the subsequent observable changes [2]. Our learners describe internal responses which are inadequately represented by “use” but significantly changed their learning. Firstly, “use” was not linear- the same learner describes using feedback and rejecting it in different contexts. Secondly, learners spend a lot of time with feedback that they do not use. They ruminate on this feedback.. Some lose confidence and motivation; some devalue feedback and the associated learning task. They take these responses with them to their future learning. These invisible responses have significantly impacted their learning values and subsequent actions.

### Implications for educators

#### Learners struggle to select strategies based on feedback

Learners find it hard to translate feedback into tangible strategies. There is an expectation in undergraduate medical students that the teacher will provide an explicit step-by-step action plan, and little consideration for learner role in co-development. Educators should foster learners’ self-regulation through encouraging incrementally more ownership of strategies, with teachers inputting where guidance is needed. Previous work indicates that facilitation in a problem-based learning (PBL) context helps learners to generate learning plans [51]. By employing reflective feedback conversations, educators can increase learner agency and engagement in self-regulation with feedback [52].

Learners may have stable learning beliefs that are a barrier to enacting feedback.

When learners have pre-existing beliefs about learning, they may be in conflict with changes advised via feedback. Facilitated reflection conversations with educators and peer and self-assessment help learners understand and appreciate their role in feedback [53]; potentially challenging preconceived notions that limit development.

#### Feedback goes unheard when learners feel unsafe

Firstly, emotional reactions are commonplace due to frequent hostility in feedback interactions, and influence subsequent attitude, engagement and learning values. Learners direct their energy to managing emotions rather than processing feedback messages, when they are distressed. While feedback literacy requires the learner to manage their emotions in navigating critique, this is distinct to handling overt hostility. Feedback-literate educators will recognise vulnerability and create a safe environment where students’ emotions are respected, allowing feedback messages to be heard.

#### Not implemented does not mean no impact

Not using feedback should not be confused with ignoring it. Learners often spend a lot of time pondering feedback. They indicate inaction is sometimes paralysis from “destroyed” confidence, sometimes devaluation of learning and learning goals. Rather than assuming indifference, we encourage educators to undertake sensitive exploration with learners to identify what may have led them to not apply feedback. This also has implications for how we evaluate “effective” feedback; a focus on facilitated reflection should be considered in addition to observable changes in the learner and their performance. A summary of our key findings and suggested approaches to supporting SRL in feedback activities is provided in Table 6.

**Table 6:** Alignment of findings with suggestions on how to support self-regulation in feedback

Findings	How to support SRL
Medical students position themselves as passive recipients of feedback Medical students show reluctance to implement feedback that requires major changes or conflicts with personal learning beliefs	Reflective conversations to support increasing awareness of students’ role in controlling their learning Reflective conversations give students insight into their learning journey, enabling them to challenge preconceptions that limit development
Medical students are junior learners whose self-regulation may not be as developed as postgraduate trainees, where models of feedback focus on co-construction of learning plans	Educators may need to initially lead learning plans to support incremental student engagement in developing learning plans
When learners feel psychologically unsafe, their ability to engage cognitively is derailed	Educators must acknowledge and support emotions in feedback conversations if they wish learners to be able engage with the feedback and use it in adapting their learning via self-regulation Educators need to communicate constructively and cultivate a supportive environment where student developmental planning is central to the agenda
When learners do not use feedback, it does not mean it has not affected them	Educators should not conflate non-implementation of feedback with no impact. Sensitive exploration of why the learner has withdrawn can support students in re-engaging

### Strengths and limitations

This study is unique in interpreting feedback responses within a theoretical framework of learning. In doing so, it aligns these responses not just with learning actions, but acknowledges the cognitive, metacognitive, effective and behavioural aspects to learning. The development of multiple iterations of the template facilitated rich understanding of interconnections and interactions between themes. Having an international research team brought a diversity of context to the interpretation. This multi-site study of students from a broad range of nationalities, with varied backgrounds and experiences, improves the likelihood of transferability of our findings. We recognise that feedback responses are complex and highly contextualised; we acknowledge that findings in postgraduate populations and other learners may be affected by such contexts. While our work elicited rich, narrative descriptions that allowed a complex analysis, we recognise that exploring learners’ internal processing may benefit from alternative data collection methods such as reflective diaries.

## Conclusions

By exploring feedback responses through the lens of SRL, we contribute to a better understanding of how learners move through feedback processing. Feedback can influence learning goals positively and negatively, depending on context. Medical students willingly adapt task-specific feedback, but continue to expect unidirectional instruction rather than the co-construction understood in modern feedback definitions. When feedback is a hostile experience, its potential is lost, as learning is derailed by distress. Conflation of non-use with indifference is cautioned; disengagement may be a sign of prolonged internal processing with significant impact on future learning.

### Supplementary Information

**Additional file 1:** **Appendix A.** Guiding Questions for Interviews.

**Additional file 2:** **Appendix B.** COREQ Checklist*.

## Data Availability

The datasets analysed in the study are available from the corresponding author on reasonable request.

## References

[1] Hattie J, Timperley H (2007). The power of feedback. Rev Educ Res.

[2] Butler DL, Winne PH (1995). Feedback and self-regulated learning: a theoretical synthesis. Rev Educ Res.

[3] Hattie J (2008). Visible learning: A synthesis of over 800 meta-analyses relating to achievement.

[4] M. Spooner et al., "Self–regulatory learning theory as a lens on how undergraduate and postgraduate learners respond to feedback: A BEME scoping review: BEME Guide No. 66," Medical Teacher, pp. 1–16, 2021. 10.1080/0142159X.2021.1970732 34666584

[5] Boud D, Molloy E (2013). Rethinking models of feedback for learning: the challenge of design. Assess Eval High Educ.

[6] H. E. F. C. f. England. " National Student Survey Results 2016. ." http://www.hefce.ac.uk/lt/nss/results/2016/ (accessed.

[7] Sinclair HK, Cleland JA. Undergraduate medical students: who seeks formative feedback?. Medical Education. 2007;41(6):580–2. 10.1111/j.1365-2923.2007.02768.x 17518838

[8] Bing-You RG, Trowbridge RL. Why medical educators may be failing at feedback. Jama. 2009;302(12):1330-1. 10.1001/jama.2009.1393 19773569

[9] Harrison CJ, Könings KD, Molyneux A, Schuwirth LW, Wass V, van der Vleuten CP. Web-based feedback after summative assessment: How do students engage?. Medical Education. 2013;47(7):734-44. 10.1111/medu.12209 23746163

[10] Winstone NE, Nash RA, Rowntree J, Parker M. ‘It'd be useful, but I wouldn't use it’: barriers to university students’ feedback seeking and recipience. Studies in Higher Education. 2017;42(11):2026-41.

[11] Ramani S, Könings KD, Ginsburg S, van der Vleuten CP (2019). Feedback redefined: principles and practice. J Gen Intern Med.

[12] Telio S, Ajjawi R, Regehr G (2015). The “educational alliance” as a framework for reconceptualizing feedback in medical education. Acad Med.

[13] Carless D, Boud D (2018). The development of student feedback literacy: enabling uptake of feedback. Assess Eval High Educ.

[14] Butler DL, Brydges R (2013). Learning in the health professions: what does self-regulation have to do with it?. Med Educ.

[15] P. R. Pintrich, "The role of goal orientation in self-regulated learning," in Handbook of self-regulation: Elsevier, 2000, pp. 451–502.

[16] Boekaerts M (1996). Self-regulated learning at the junction of cognition and motivation. Eur Psychol.

[17] B. J. Zimmerman and D. H. Schunk, Self-regulated learning and academic achievement: Theoretical perspectives. Routledge, 2001.

[18] Ashford SJ, Blatt R, Walle DV (2003). Reflections on the looking glass: a review of research on feedback-seeking behavior in organizations. J Manag.

[19] Carless D (2019). Feedback loops and the longer-term: towards feedback spirals. Assess Eval High Educ.

[20] Sargeant J (2015). Facilitated reflective performance feedback: developing an evidence-and theory-based model that builds relationship, explores reactions and content, and coaches for performance change (R2C2). Acad Med.

[21] Garino A (2020). Ready, willing and able: a model to explain successful use of feedback. Adv Health Sci Educ.

[22] Varpio L, MacLeod A (2020). Philosophy of science series: harnessing the multidisciplinary edge effect by exploring paradigms, ontologies, epistemologies, axiologies, and methodologies. Acad Med.

[23] Tong A, Sainsbury P, Craig J (2007). Consolidated criteria for reporting qualitative research (COREQ): a 32-item checklist for interviews and focus groups. Int J Qual Health Care.

[24] Finlay L (2002). Negotiating the swamp: the opportunity and challenge of reflexivity in research practice. Qual Res.

[25] Watt D (2007). On becoming a qualitative researcher: the value of reflexivity. Qualitative Report.

[26] F. M. Olmos-Vega, R. E. Stalmeijer, L. Varpio, and R. Kahlke, "A practical guide to reflexivity in qualitative research: AMEE Guide No. 149," Medical Teacher, pp. 1–11, 2022. 10.1080/0142159X.2022.2057287 35389310

[27] RCSI. "https://www.rcsi.com/education/international-outlook." Accessed 3 June 2023.

[28] Malterud K, Siersma VD, Guassora AD (2016). Sample size in qualitative interview studies: guided by information power. Qual Health Res.

[29] Brooks J, McCluskey S, Turley E, King N (2015). The utility of template analysis in qualitative psychology research. Qual Res Psychol.

[30] King N (2012). Doing template analysis," Qualitative organizational research. Core Methods Curr Challenges.

[31] Creswell JW, Creswell JD (2017). Research design: qualitative, quantitative, and mixed methods approaches. Sage Pub.

[32] Sargeant J (2017). R2C2 in action: testing an evidence-based model to facilitate feedback and coaching in residency. J Grad Med Educ.

[33] Ajjawi R, Regehr G (2019). When I say… feedback. Med Educ.

[34] Berkhout JJ, Teunissen PW, Helmich E, van Exel J, van der Vleuten CP, Jaarsma DA (2017). Patterns in clinical students’ self-regulated learning behavior: a Q-methodology study. Adv Health Sci Educ.

[35] Burke D (2009). Strategies for using feedback students bring to higher education. Assess Eval High Educ.

[36] Kornell N, Bjork RA (2008). Optimising self-regulated study: The benefits—and costs—of dropping flashcards. Memory.

[37] Cho KK, Marjadi B, Langendyk V, Hu W (2017). The self-regulated learning of medical students in the clinical environment–a scoping review. BMC Med Educ.

[38] Berkhout JJ, Helmich E, Teunissen PW, van der Vleuten CP, Jaarsma ADC (2017). How clinical medical students perceive others to influence their self-regulated learning. Med Educ.

[39] Schommer M (1990). Effects of beliefs about the nature of knowledge on comprehension. J Educ Psychol.

[40] O’Donovan B (2017). How student beliefs about knowledge and knowing influence their satisfaction with assessment and feedback. High Educ.

[41] Evensen DH, Salisbury-Glennon JD, Glenn J (2001). A qualitative study of six medical students in a problem-based curriculum: toward a situated model of self-regulation. J Educ Psychol.

[42] Sargeant J, Mann K, Sinclair D, Van der Vleuten C, Metsemakers J (2008). Understanding the influence of emotions and reflection upon multi-source feedback acceptance and use. Adv Health Sci Educ.

[43] Eva KW, Armson H, Holmboe E, Lockyer J, Loney E, Mann K, Sargeant J (2012). Factors influencing responsiveness to feedback: on the interplay between fear, confidence, // reasoning processes. Adv Health Sci Educ.

[44] Mann K (2011). Tensions in informed self-assessment: how the desire for feedback // reticence to collect // use it can conflict. Acad Med.

[45] McKenzie J, Olson R, Patulny R, Peterie M (2019). "Social emotions: a multidisciplinary approach," emotions: history. Culture, Society.

[46] Pekrun R (2006). The control-value theory of achievement emotions: assumptions, corollaries, and implications for educational research and practice. Educ Psychol Rev.

[47] I. M. Nembhard and A. C. Edmondson, "Psychological safety," in The Oxford handbook of positive organizational scholarship, 2012.

[48] Zhang J-Y, Liu Y-J, Shu T, Xiang M, Feng Z-C (2022). Factors associated with medical students’ self-regulated learning and its relationship with clinical performance: a cross-sectional study. BMC Med Educ.

[49] Ajjawi R, Olson RE, McNaughton N (2022). Emotion as reflexive practice: a new discourse for feedback practice and research. Med Educ.

[50] Orsmond P (2005). Biology students’ utilization of tutors’ formative feedback: a qualitative interview study. Assess Eval Higher Educ.

[51] Mubuuke A, Louw A, Van Schalkwyk S (2017). Self-regulated learning: a key learning effect of feedback in a problem-based learning context. Afr J Health Prof Educ.

[52] Ducasse AM, Hill K (2019). Developing student feedback literacy using educational technology and the reflective feedback conversation. Practitioner Res Higher Educ.

[53] T. Little, P. Dawson, D. Boud, and J. Tai, "Can students’ feedback literacy be improved? A scoping review of interventions," Assessment & Evaluation in Higher Education, 2023;1–14,.

