# Supplementary material for: “Tell me what is ‘better’!” How medical students experience feedback, through the lens of self-regulatory learning
Source: BMC Med Educ. 2023 Nov 22;23:895. doi: 10.1186/s12909-023-04842-9 (PMC10666439; doi:10.1186/s12909-023-04842-9)
Supplement: Supplementary file 1 — Additional file 1: Appendix A. Guiding Questions for Interviews. [file 12909_2023_4842_MOESM1_ESM.docx]

# Appendix A.

## Guiding Questions for Interviews

1. Tell me what you consider to be feedback
2. What do you consider to be feedback?
3. Tell me about your feedback experiences before medical school.
4. Do you seek feedback? Tell me about this.
5. If not, then why not?
6. What would it take for you to seek out feedback?
7. What motivates you to seek feedback? What are your goals when receiving feedback?
8. Tell me about how feedback affects what learning strategies you use.
9. Tell me about how feedback affects how you implement/organise your learning?
10. How do you check if applying feedback has helped your learning?
11. Do you think your life experience affects your attitude to feedback?

Questions are based on Pintrich’s model of self-regulation (1. Forethought, planning and activation; 2. Monitoring; 3. Control; 4 Reflection and Reaction). Pintrich, P. R. (2000). The role of goal orientation in self-regulated learning. In *Handbook of self-regulation* (pp. 451-502).
