# Supplementary material for: “Tell me what is ‘better’!” How medical students experience feedback, through the lens of self-regulatory learning
Source: BMC Med Educ. 2023 Nov 22;23:895. doi: 10.1186/s12909-023-04842-9 (PMC10666439; doi:10.1186/s12909-023-04842-9)
Supplement: Supplementary file 2 — Additional file 2: Appendix B. COREQ Checklist*. [file 12909_2023_4842_MOESM2_ESM.docx]

# Appendix B

## COREQ Checklist*

| **No. Item** | **Guide questions/description** | **Reported on Page #** |
| --- | --- | --- |
| **Domain 1: Research team and reﬂexivity** |  |  |
| *Personal Characteristics* |  |  |
| 1. Inter viewer/facilitator | Which author/s conducted the interview or focus group? | MHJ,JL,MS  Page 7, paragraph 1 |
| 2. Credentials | What were the researcher’s credentials? E.g. PhD, MD | MS- MBBCHBAO, PG Dip HPE  TP-MB BS MSc PhD MRCP DRCOG FRCPI  JL-  MHJ- BSC, PhD  CSL-MB BS MSc  SM- MD, FRCP, FRCPI, AM, DTM&H  Page 5, paragraph 2 |
| 3. Occupation | What was their occupation at the time of the study? | MS- senior lecturer  TP- Director HPEC  JL- doctoral researcher  MHJ- Post-doctoral researcher  CSL- Associate Professor GP  SM- Professor of ID |
| 4. Gender | Was the researcher male or female? | MS- female  TP- female  JL- male  MHJ- male  CSL- female  SM- male |
| 5. Experience and training | What experience or training did the researcher have? | All experienced qualitative researchers. MS in final stages of PhD and has completed PG Dip in HPE |
| *Relationship with participants* |  |  |
| 6. Relationship established | Was a relationship established prior to study commencement? | No, interviewers introduced themselves to participants at time of interviews |
| 7. Participant knowledge of the interviewer | What did the participants know about the researcher? e.g. personal goals, reasons for doing the research | Participants were briefed on the purpose of the study and understood it.  Page 5, paragraph 2  Ethics had granted, participants reviewed the participant information documentation prior to giving their written informed consent to be involved. |
| 8. Interviewer characteristics | What characteristics were reported about the interviewer/facilitator? e.g. Bias, assumptions, reasons and interests in the research topic | MS regularly provides feedback to students, and is completing a PhD on feedback, which is a potential source of bias. No other sources of bias were identified. |

| **Domain 2: study design** |  |  |
| --- | --- | --- |
| *Theoretical framework* |  |  |
| 9. Methodological orientation and Theory | What methodological orientation was stated to underpin the study? e.g. grounded theory, discourse analysis, ethnography, phenomenology, content analysis | Socio-constructivist epistemology, template analysis of interviews, through lens of SRL theory  Page 5, paragraph 1 |
| *Participant selection* |  |  |
| 10. Sampling | How were participants selected? e.g. purposive, convenience, consecutive, snowball | Purposive, Page 6, paragraph 1 |
| 11. Method of approach | How were participants approached? e.g. face-to-face, telephone, mail, email | Virtual learning environment notice and face-to-face communication to explain study. Participants then emailed expression of interest to gatekeeper to arrange interview  Page 6, paragraph 1 |
| 12. Sample size | How many participants were in the study? | 57 |
| 13. Non-participation | How many people refused to participate or dropped out? Reasons? | None |
| *Setting* |  |  |
| 14. Setting of data collection | Where was the data collected? e.g. home, clinic, workplace | Meeting room in Health Professions Education Centre, Dublin. Online for Manama (Bahrain) and Perdana (Malaysia) |
| 15. Presence of non-participants | Was anyone else present besides the participants and researchers? | No |
| 16. Description of sample | What are the important characteristics of the sample? e.g. demographic data, date | Table 2 |
| *Data collection* |  |  |
| 17. Interview guide | Were questions, prompts, guides provided by the authors? Was it pilot tested? | Yes. Interviews were semi-structured using a guide (Appendix A), follow-up questions were allowed  Yes, pilot-testing details page 6, paragraph 1 |
| 18. Repeat interviews | Were repeat interviews carried out? If yes, how many? | No |
| 19. Audio/visual recording | Did the research use audio or visual recording to collect the data? | Audio recordings |
| 20. Field notes | Were ﬁeld notes made during and/or after the interview or focus group? | Yes, after the interviews |
| 21. Duration | What was the duration of the inter views or focus group? |  |
| 22. Data saturation | Was data saturation discussed? |  |
| 23. Transcripts returned | Were transcripts returned to participants for comment and/or correction? | No |
| **Domain 3: analysis and ﬁndings** |  |  |
| *Data analysis* |  |  |
| 24. Number of data coders | How many data coders coded the data? | MS and TP |
| 25. Description of the coding tree | Did authors provide a description of the coding tree? | Page 7 / paragraph 2 (Line 112) |
| 26. Derivation of themes | Were themes identiﬁed in advance or derived from the data? | *A priori* themes were developed from SRL theory. Subsequent themes were derived from the data. Themes were compared, edited, deleted when not relevant to the RQ, and re-arranged hierarchically.  Page 7 |
| 27. Software | What software, if applicable, was used to manage the data? | Microsoft Word, Excel, N Vivo |
| 28. Participant checking | Did participants provide feedback on the ﬁndings? | Yes |
| *Reporting* |  |  |
| 29. Quotations presented | Were participant quotations presented to illustrate the themes/ﬁndings? Was each quotation identiﬁed? e.g. participant number | Yes |
| 30. Data and ﬁndings consistent | Was there consistency between the data presented and the ﬁndings? | Yes |
| 31. Clarity of major themes | Were major themes clearly presented in the ﬁndings? | Yes, themes were supported with direct quotes attributed to anonymised participants |
| 32. Clarity of minor themes | Is there a description of diverse cases or discussion of minor themes? | Yes, minor and diverse themes are discussed in the manuscript |

*Consolidated criteria for reporting qualitative studies (COREQ): 32-item checklist

Developed from: Tong A, Sainsbury P, Craig J. Consolidated criteria for reporting qualitative research (COREQ): a 32-item checklist for interviews and focus groups. *International Journal for Quality in Health Care*. 2007. Volume 19, Number 6: pp. 349 – 357
